# Impact of middle and lower jugular neck dissection on supraclavicular lymph node metastasis from endometrial carcinoma

**DOI:** 10.1186/1477-7819-10-143

**Published:** 2012-07-12

**Authors:** Masataka Kojima, Junkichi Yokoyama, Shin Ito, Shinichi Ohba, Mitsuhisa Fujimaki, Katsuhisa Ikeda

**Affiliations:** 1 Department of Otorhinolaryngology Head and Neck Surgery, Juntendo University Faculty of Medicine, 2-1-1, Hongo, Bunkyo-ku, Tokyo, Japan, 113-8421

**Keywords:** Neck dissection, Thoracic duct, Cervical lymph node, Endometrial adenocarcinoma, Chyle fistula

## Abstract

Supraclavicular lymph node metastasis from endometrial carcinoma is considerably rarer than metastasis from uterine cervical cancer. To date, there have been no reported cases regarding systematic neck dissection as a salvage treatment. In this report, we describe the neck dissection procedure carried out on a 74-year-old woman with supraclavicular lymph node metastasis. Our objective was to histologically determine the origin of the metastasis while simultaneously providing appropriate treatment. The patient’s past medical history included two prior cases of cancer: rectal cancer 7 years earlier and endometrial adenocarcinoma 4 years earlier. We determined that middle and lower jugular neck dissection was appropriate in treating this case based on the results of our preoperative FDG-PET and tumor markers. This surgery provided histological evidence that metastasis occurred from endometrial carcinoma. Middle and lower jugular neck dissection was expected to improve the patient’s prognosis without impacting the patient’s active daily life. We have continued to monitor the patient closely over an extended period.

## Background

Supraclavicular lymph node metastasis from uterine carcinoma is rare and has been shown to negatively affect a patient’s prognosis. If supraclavicular lymph node metastases is detected from cervical cancer of the uterine, there is the possibility of further distant metastasis occurring. Therefore, the prognosis of supraclavicular lymph node metastasis from uterine cervical cancer is considered significant. In such cases, palliative radiotherapy is the recommended treatment for relief of symptoms and improvement of the patient’s quality of life.

Metastases of uterine carcinoma to the neck is reported to spread by way of the lymph flow from the pelvis up to the para-aortic nodes into the mediastinum, then into the thoracic duct [1]. There have been no cases reported regarding the use of systematic neck dissection for the treatment of left supraclavicular lymph nodes suspected of being endometrial adenocarcinoma metastasis before surgery. Neck dissection for the treatment of metastasis from endometrial adenocarcinomas is not an established procedure.

The objective of our report is to consider the most effective method of neck dissection for the treatment of supraclavicular lymph nodes from endometrial carcinoma in relation to our patient.

## Case presentation

**Figure 1:**
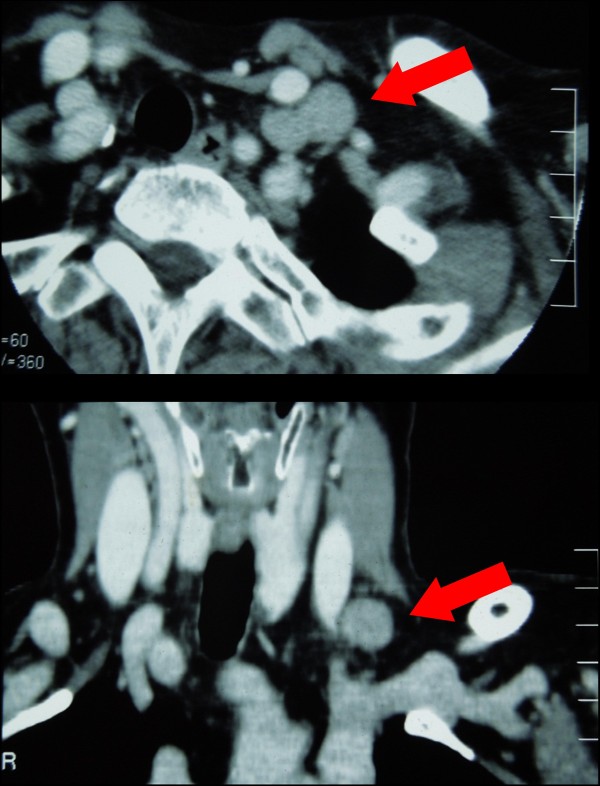
**Preoperative CT findings.** The CT showed a single left supraclavicular lymph node (arrow) (**a**) axial section, (**b**) coronal section

**Figure 2:**
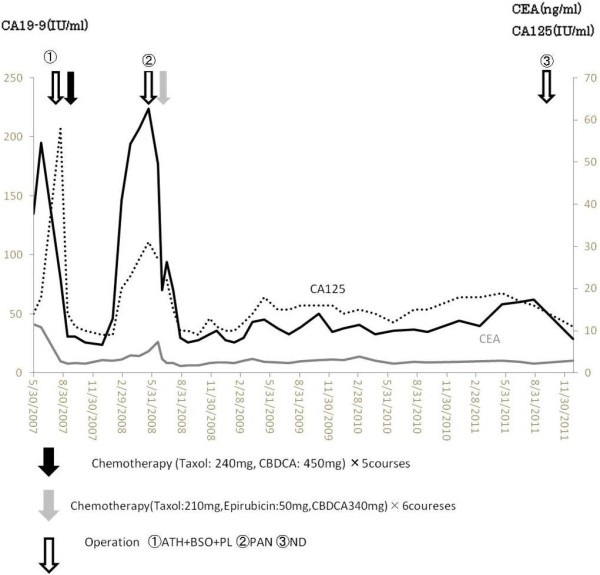
**Changes of tumor markers (CA19-9, CA125, and CEA).** The course of the treatment for endometrial carcinoma and the changes of tumor markers

A 74-year-old woman underwent computed tomography (CT) as a follow-up procedure following rectal cancer. In the findings, we detected a left supraclavicular lymph node suspected of metastasis (Figure 1). The patient’s past medical history includes the following: 7 years earlier she underwent rectal cancer treatment with low anterior resection and postoperative chemotherapy; 4 years earlier she underwent a total hysterectomy, bilateral salpingo-oophorectomy and pelvic lymphadenectomy for a stage IIIc endometrial adenocarcinoma (pT1cN1M0). Figure 2 shows the changes of tumor markers (CA19-9, CA125, and CEA) from the time of diagnosis to the present. Subsequent to the operation, the patient received adjuvant chemotherapy. Approximately 1 year later, the patient underwent para-aortic lymphadenectomy and additional chemotherapy for the recurrence in the para-aortic lymph node of endometrial adenocarcinoma. There was no clinical evidence of local or distant recurrence from either of the two malignancies at follow-up.

**Figure 3:**
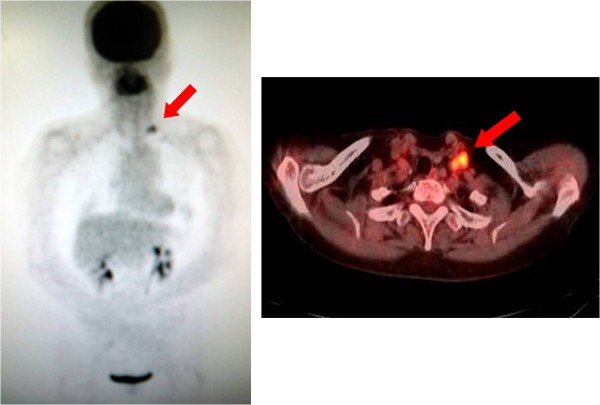
**The positron emission tomography-computed tomography (PET-CT).** PET-CT showed a single left supraclavicular lymph node (the arrow) with high uptake (SUV max 6.0). There were no other metastatic lesions in the body

**Figure 4:**
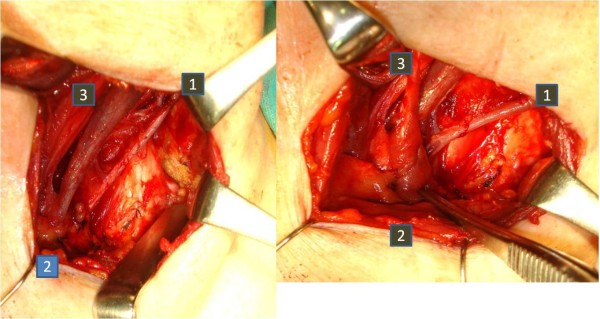
**Intraoperative findings.** (**a**) Thoracic duct was resected with supraclaviclar metastatic lymph nodes. Each number shows: (1) external jugular vein; (2) resected thoracic duct; (3) internal jugular vein. (**b**) The inferior belly of the omohyoid muscle was cut distally and tightly sutured on the venous angle. (1) External jugular vein; (2) inferior belly of omohyoid muscle augmented to resected thoracic duct; (3) superior belly of omohyoid muscle

Fine needle aspiration biopsy of the left supraclavicular lymph node showed adenocarcinoma metastasis. Positron emission tomography-computed tomography (PET-CT) revealed high uptake (SUVmax 6.0) in the left supraclavicular lymph node but no other metastatic lesions elsewhere (Figure 3). Middle and lower jugular neck dissection was performed in 2011 on the grounds that volume reduction would contribute to the improvement of prognosis, and residual metastasis was only identified in the left supraclavicular lymph node. A skin incision was made 2 cm above the left clavicle. Some solid lymph nodes could be palpated between the left sternocleidomastoid muscle’s sternal head and clavicular head. The thoracic duct was also resected with the supraclavicular metastatic lymph nodes (Figure 4a). There was no lymphorrhea from the juglosubclavian angle. To prevent chyle fistula, the inferior belly of the omohyoid muscle was cut distally and tightly sutured on the venous angle (Figure 4b). The sternocleidomastoid muscle’s sternal head was obliterated into the dead space of the venous angle with absorbable sutures. A continuous suction drainage tube was inserted into the supraclavicular space. Surgical time was 49 min and blood loss during surgery was 10 mL. Following the operation the amount of drainage was slight and the drainage tube was removed on the second postoperative day. The patient left hospital on the fifth postoperative day without any complications such as dysfunction of the upper limbs or shoulders.

**Figure 5:**
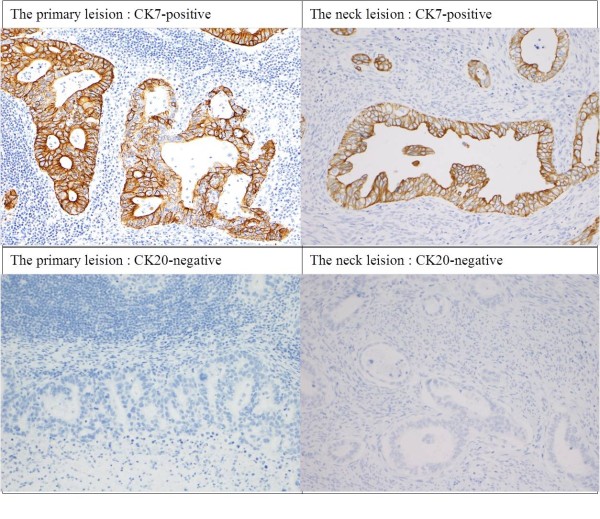
**Immunohistchemical staining examination.** Immunohistchemical staining examination using cytokeratin7 and cytokeratin20 indicated that the neck metastasis originated from endometrial adenocarcinoma

A histological examination showed that there was no metastasis among the six dissected lymph nodes at level III, and only one metastasis was detected among the 11 dissected lymph nodes at level IV. An immunohistochemical staining examination using cytokeratin 7 and cytokeratin 20 was performed in order to diagnose the primary disease and determine whether it was rectal cancer or endometrial cancer. The immunohistochemical staining examination indicated that the neck metastasis originated from endometrial adenocarcinoma [2,3] (Figure 5).

Extracapsular spread was not detected pathologically and consequently the patient received no additional adjuvant treatment. The patient now lives a normal life without recurrence.

## Discussion

Cervical lymph node metastases from genito-urinary neoplasms are rare. Left-sided supraclavicular metastases are predominant because of the anatomy of the lymphatic system [1,4]. Oosaki *et al*. reported that there were only five patients (0.15%) with neck metastasis of endometrial cancer in a total number of 3,230 patients with neck metastasis of gynecological malignancy [5]. In cervical cancer of the uterine, however, supraclavicular lymph nodes sampling has been performed as part of the pretreatment evaluation, especially in cases in which extended field radiation was planned. The overall incidence of occult metastasis in the supraclavicular nodes from cervical cancer of uterine was 15.5% (55/359) [6]. When the supraclavicular lymph nodes are involved with metastasis from cervical cancer of the uterine, there is a possibility of metastasis to the para-aortic lymph nodes, lungs, liver, and peritoneal cavity [1,6,7]. The prognosis of supraclavicular lymph nodes metastasis in cervical cancer of the uterine is considered very severe. The 3-year and 5-year overall survival rates indicated within several studies were recorded between 0% to 28% [1,7,8]. Once supraclavicular node metastasis is detected, cure is not the objective of treatment, but rather palliative measures for relief of symptoms and improving the patients’ quality of life [1,7,8].

However, there have been very few case reports documenting neck dissection for the treatment of neck metastasis of endometrial cancer. Yoshitake *et al*. reported supraclavicular lymph node metastasis originating from cancer of uterine body incidentally detected following neck dissection carried out during the treatment of a patient with upper gingival cancer [9]. Siddiq *et al.* also reported on a patient with neck metastasis from laryngeal carcinoma, breast carcinoma, and endometrial carcinoma. A pathological examination from a resected specimen demonstrated a clear cell endometrial carcinoma originated from endometrial carcinoma [10]. However, there has been no report of systematic neck dissection for the treatment of neck lesion from endometrial cancer before a preoperative diagnosis. Volume reduction surgery for the treatment of recurrent endometrial cancer has been considered to be effective. Anderson *et al*. reported that if the metastasis was localized in the lung, the resection of the pulmonary metastasis would improve the prognosis [11].

In our case, we chose middle and lower jugular neck dissection as a treatment and diagnosis for the supraclavicular metastasis of endometrial carcinoma after first considering resectability, patient’s age, performance status, post-treatment quality of life, hospitalization, postoperative shoulder function, radiation resistibility [12], and drug resistibility [13,14].

A neck dissection method for treating endometrial adenocarcinoma metastasis to the neck has not yet been established. If there is no extranodal invasion in the neck lymph nodes, we consider middle and lower jugular neck dissection for the apparent metastasis level to be a beneficial therapeutic procedure. Left-sided supraclavicular metastasis is predominant and is transferred via the thoracic duct [4]. In our case, supraclavicular lymph nodes metastases were palpable in the single level IV, and so middle and lower jugular neck dissection was performed for levels III and IV.

Postoperative cervical chyle fistula is rare, however once it occurs it is difficult to control after neck dissection. Chyle fistula causes electrolyte and protein loss, wound infection, carotid blowout, chylethorax, and fatal anoxia. It also delays postoperative treatment such as radiotherapy and chemotherapy [15]. Consequently it is necessary to prevent the chyle fistula intraoperatively. Postoperative chyle fistula occurs at a rate of 1% to 3% in left side radical neck dissections [15]. However, tying the damaged duct closure is not always an easy procedure. The wall of the thoracic duct is often extremely thin and over-sewing often increases leakage conversely [16]. As a result, using muscle flaps is reported to be an effective method of preventing chyle fistula [15-17]. In addition, the benefits of surgical closure of leakage with clavicular periosteal flap, or pectoralis major muscle flap used with fibrin glue have been reported [15,16,18].

The technique using the omohyoid muscle flap is straightforward and a reliable procedure for preventing chyle fistula over a short period. By cutting the inferior belly of the muscle distally and transferring it to the site of the juglosubclavian angle, the vascularized pedicle muscle belly flap does not result in any negative functional or esthetic consequences for the patient [15].

The most important factor influencing the radiosensitivity of endometrial carcinoma is the duration of menopause. The older the patient’s age, the worse the radiosensitivity for endometrial carcinoma [12]. Once platinum- and taxane-based chemotherapy consistently demonstrated the highest response in the treatment of endometrial carcinoma, however, in recurrent cases, the endometrial carcinoma is often platinum = and taxane-resistant [13,14] For this reason, we selected surgical treatment for neck metastasis rather than radiotherapy due to sound control of the disease and sound pathological diagnosis of the disease. If a single neck metastasis after chemotherapy was resectable, we recommend surgical treatment with advantages including good quality of life, and short hospitalization without postoperative complications. However, a standard procedure for neck dissection has not been established for neck metastasis originating from endometrial cancer. Further clinical studies of the neck dissection for gynecological malignancy including endometrial cancer are required.

## Conclusions

The best procedure for carrying out neck dissection for treating neck metastases from endometrial cancer has not been established to date. Middle and lower jugular neck dissection enables pathological diagnosis and improves the patient’s prognosis without any postoperative complications. Furthermore, the technique of using the omohyoid muscle flap is very useful for preventing chyle fistula.

## Consent

Written informed consent was obtained from the patient for publication of this case report and all accompanying images. A copy of the written consent is available for review by the Editor-in-Chief of this journal.

## Abbreviations

CT, Computed tomography; PET-CT, Positron emission tomography-computed tomography; SUVmax, Maximum 18 F-FDG standardized uptake value.

## Competing interests

The authors declare that they have no competing interests.

## Authors’ contributions

JY carried out the initial conception and design as well as collection of data and clinical records of the patient. MK participated in its design and helped to edit the manuscript. SI and SO made up the surgical team involved in the case and carried out the initial conception. MF and KI assisted to revise the manuscript. All authors read and approved the final manuscript.
